# Novel Therapies for Alport Syndrome

**DOI:** 10.3389/fmed.2022.848389

**Published:** 2022-04-25

**Authors:** Efren Chavez, Juanly Rodriguez, Yelena Drexler, Alessia Fornoni

**Affiliations:** ^1^Katz Family Division of Nephrology and Hypertension, Department of Medicine, University of Miami Miller School of Medicine, Miami, FL, United States; ^2^Peggy and Harold Katz Family Drug Discovery Center, University of Miami Miller School of Medicine, Miami, FL, United States

**Keywords:** Alport, Alport disease, Alport nephritis, Alport hereditary nephritis, COL4, type IV collagen, collagen 4, treatment

## Abstract

Alport syndrome (AS) is a hereditary kidney disease associated with proteinuria, hematuria and progressive kidney failure. It is characterized by a defective glomerular basement membrane caused by mutations in type IV collagen genes *COL4A3/A4/A5* which result in defective type IV collagen α3, α4, or α5 chains, respectively. Alport syndrome has three different patterns of inheritance: X-linked, autosomal and digenic. In a study of CKD of unknown etiology type IV collagen gene mutations accounted for the majority of the cases of hereditary glomerulopathies which suggests that AS is often underrecognized. The natural history and prognosis in patients with AS is variable and is determined by genetics and environmental factors. At present, no preventive or curative therapies exist for AS. Current treatment includes the use of renin-angiotensin-aldosterone system inhibitors which slow progression of kidney disease and prolong life expectancy. Ramipril was found in retrospective studies to delay the onset of ESKD and was recently demonstrated to be safe and effective in children and adolescents, supporting that early initiation of Renin Angiotensin Aldosterone System (RAAS) blockade is very important. Mineralocorticoid receptor blockers might be favorable for patients who develop “aldosterone breakthrough.” While the DAPA-CKD trial suggests a beneficial effect of SGLT2 inhibitors in CKD of non-metabolic origin, only a handful of patients had Alport in this cohort, and therefore conclusions can't be extrapolated for the treatment of AS with SGLT2 inhibitors. Advances in our understanding on the pathogenesis of Alport syndrome has culminated in the development of innovative therapeutic approaches that are currently under investigation. We will provide a brief overview of novel therapeutic targets to prevent progression of kidney disease in AS. Our review will include bardoxolone methyl, an oral NRf2 activator; lademirsen, an anti-miRNA-21 molecule; sparsentan, dual endothelin type A receptor (ETAR) and angiotensin 1 receptor inhibitor; atrasentan, oral selective ETAR inhibitor; lipid-modifying agents, including cholesterol efflux transporter ATP-binding cassette A1 (ABCA1) inducers, discoidin domain receptor 1 (DDR1) inhibitors and osteopontin blocking agents; the antimalarial drug hydroxychloroquine; the antiglycemic drug metformin and the active vitamin D analog paricalcitol. Future genomic therapeutic strategies such as chaperone therapy, genome editing and stem cell therapy will also be discussed.

## Introduction

Alport syndrome (AS), also called hereditary nephritis, is an inheritable progressive glomerular disease that is generally associated with sensorineural hearing loss and ocular abnormalities. It presents with structural anomalies and dysfunction of the glomerular basement membrane (GBM) caused by genetic mutations affecting the type IV collagen α3/α4/α5 chains (1). Type IV collagen is essential for GBM stability and constitutes the majority of total GBM protein mass. The α1 to α6 type IV collagen chains are genetically different and they assemble to form 3 distinct heterotrimers: α1α1α2, α3α4α5 and α5α5α6 (2). In AS, pathogenic variants in the genes *COL4A3* and *COL4A4* (which are located on chromosome 2) and *COL4A5* (which is located on the X chromosome) produce defective type IV collagen α3, α4 or α5 chains respectively, which hampers the appropriate assembly of the GBM (3, 4).

## Classification

The new classification scheme categorizes AS into 3 different types based on the affected type IV collagen genes involved and mode of inheritance: X-linked AS, autosomal or digenic (Table 1).

**Table 1:** New classification system of Alport syndrome.

**Inheritance**	**Affected gene (s)**	**Allelic state**	**Mutation phenotype**
X-linked	*COL4A5*	Heterozygous (males)	NA
		Heterozygous (females)	NA
Autosomal	*COL4A3* or *COL4A4*	Homozygous or compound heterozygous	Recessive
		Heterozygous	Dominant
Digenic	*COL4A3, COL4A4* and *COL4A5*	Variable	

*NA, not applicable Adapted data from Kashtan et al. (3)*.

According to this classification system it is not necessary to have a positive family history, extrarenal manifestations or evidence of progressive kidney disease to make a diagnosis of AS. Females with heterozygous *COL4A5* variants are classified as having AS instead of being labeled as “carriers” of the disease. Patients with isolated microscopic hematuria and heterozygous variants in *COL4A3* or *COL4A4* are considered to have autosomal dominant Alport syndrome (ADAS), which therefore eliminates the diagnosis of thin basement membrane nephropathy (TBMN) (3). It must be recognized that this latter point is controversial among experts on this condition and this is not a universal agreement (5).

Genetic testing in AS is proposed for patients who have persistent dysmorphic hematuria for longer than 6 months; persistent proteinuria >0.5 g/g, family history of hematuria or kidney disease; sensorineural hearing loss with hematuria; lenticonus, fleck retinopathy or temporal retinal thinning; biopsy-proven focal segmental glomerulosclerosis (FSGS) or steroid-resistant nephrotic syndrome; GBM lamellation; chronic kidney disease of unknown etiology with hematuria and familial IgA glomerulonephritis. Genetic testing is also recommended in first-degree relatives of people with known pathogenic variants in *COL4A3-COL4A5* genes (6).

## Prevalence

The prevalence of Alport syndrome varies greatly in different reports with estimates ranging from one in 5,000 to one in 53,000 patients with kidney disease. X-linked Alport syndrome (XLAS) is caused by *COL4A5* gene mutations and accounts for 70–80% of patients with AS. Autosomal recessive Alport syndrome (ARAS) is caused by homozygous or compound heterozygous mutations in *COL4A3* and *COL4A4* genes and accounts for ~5% of patients with AS. A recent study estimated the prevalence of predicted pathogenic *COL4A3-COL4A5* variants in populations without kidney disease. Predicted pathogenic *COL4A5* variants were found in one in 2,320 persons. Predicted pathogenic heterozygous *COL4A3* and *COL4A4* variants affected one in 106 individuals, consistent with the finding of TBMN in kidney biopsies in donors without known kidney disease. Predicted pathogenic compound heterozygous variants appeared in one in 88,866 persons and digenic variants in at least one in 44,793 persons. However, these population frequencies of predicted *COL4A3-COL4A5* pathogenic variants must be modified according to the disease penetrance of individual variants (7).

The true prevalence of AS is likely underestimated. A recent study from Columbia University using genetic testing to evaluate cases of chronic kidney disease of unknown etiology showed that Mendelian nephropathies accounted for 21% of the cases. Monogenic glomerulopathies were the most frequent and this was mainly driven by mutations in collagen IV genes which accounted for nearly one third of all hereditary kidney diseases (8).

## Natural History and Prognosis

AS encompasses a diverse phenotypic spectrum with a broad range of clinical manifestations. The typical kidney histological lesion in a patient with established AS is described on electron microscopy as irregular thinning and thickening GBM with lamellated appearance which has a characteristic “basket-weave” pattern (9). When a kidney biopsy performed in a patient with isolated hematuria shows exclusively a thin GBM it has been proposed to use the terms “benign familial hematuria” or “thin basement membrane nephropathy.” However, this is an erroneous assumption since there is growing evidence that some of these patients are at risk of developing progressive kidney disease. In patients with AS, a thin GBM can also be identified and therefore this histologic finding should not be considered a separate disorder. A thin GBM by itself is not sufficient to estimate prognosis in the absence of additional clinical, pathologic, pedigree and genetic data.

The likelihood of kidney disease progression in patients affected with AS is variable and is mainly determined by the type of gene mutation. Large deletions, nonsense mutations and small mutations affecting reading frames that cause a decreased or absent functional protein represent a high risk of progression to end stage kidney disease (ESKD) by age 30. The risk is minor with missense or splice site mutations (10). Also, environmental factors may contribute to kidney disease progression.

Men with XLAS and all patients with ARAS typically develop “classic” AS features which include progressive kidney disease with hematuria and proteinuria, sensorineural deafness and ocular abnormalities (eg, lenticonus or maculopathy). While less recognized as a typical syndromic feature, aortic aneurisms are often recognized in males with XLAS (11).

In cases of XLAS, sex is an important prognostic factor regarding CKD progression. Among untreated men with XLAS, around 50 and 90% progress to ESKD by ages 25 and 40, respectively. Women with XLAS exhibit a variable phenotype due to the process of X chromosome inactivation. Among women with XLAS, about 12% reach ESKD before age 40 and this proportion increases to about 30% by age 60 and 40% by age 80 (10). Patients with ARAS frequently progress to ESKD by age 40 regardless of sex (12).

Patients with ADAS exhibit a wide variety of manifestations which range from asymptomatic to kidney-limited disease with microscopic hematuria or proteinuria and in some cases progression to ESKD. The phenotype associated with a heterozygous mutation in *COL4A3* or *COL4A4* can vary significantly even within members of the same family, and this variability of expression is likely multifactorial. This incomplete penetrance may be related to the presence of modifier genes that ameliorate or exacerbate the effects of type IV collagen gene mutations and other factors such as high blood pressure, high sodium diet, obesity and smoking which may independently contribute to kidney disease (3, 13).

In patients with ADAS, the estimated risk for ESKD is ≥20% for those with risk factors for progression (proteinuria, FSGS, GBM thickening and lamellation, sensorineural hearing loss, genetic modifiers) and <1% in the absence of these risk factors (3).

The estimated risk for progression to ESKD in patients with digenic AS is variable and depends on the affected genes: for *COL4A3* and *COL4A4* mutations in trans simulating autosomal recessive inheritance the risk is up to 100%; for *COL4A3* and *COL4A4* mutations in cis simulating autosomal dominant inheritance the risk is up to 20% and for affected men with mutations in *COL4A5* and either *COL4A3* and *COL4A4* the risk is up to 100% (3).

Type IV collagen gene mutations have also been associated with FSGS and genetic testing should be considered particularly in those patients with positive family history of FSGS and younger age at presentation. Type IV collagen gene mutations were discovered in 38% of patients with familial FSGS and 3% with sporadic FSGS with more than half of the mutations appearing in *COL4A5*. The presence of hematuria, hearing loss and GBM abnormalities might indicate the possibility of an underlying *COL4* mutation (14). In a cohort of 193 individuals of the Toronto GN registry with predominantly sporadic FSGS who underwent whole-exome sequencing, the genetic diagnostic rate was 11% and from these patients 55% had definitely pathogenic variants in *COL4 (A3/A4/A5)* genes (15). Individuals with biopsy-proven FSGS who have pathogenic variants in the type IV collagen genes should be classified as Alport syndrome patients. It is important to avoid the use of immunosuppressive therapy on these patients because this would be ineffective and potentially harmful. Consistent with these observations, genetic testing should be obtained in all patients with biopsy-proven FSGS or steroid resistant nephrotic syndrome (6).

## Kidney Transplantation

Patient with Alport syndrome who progress to ESKD are generally excellent candidates for kidney transplantation. Preemptive kidney transplantation should be pursued when possible. In patients with AS, graft survival rates are equal or better compared to those seen in patients with other causes of ESKD. It is recommended that family members who are *COL4A3* and *COL4A4* heterozygotes not be considered for kidney donation (6). There is evidence from individuals with hematuria or heterozygous pathogenic *COL4A3* or *COL4A4* variants who acted as kidney donors who developed worsening kidney function or proteinuria over time (16–18). While the development of anti-GBM antibodies without clinical manifestation is quite common, posttransplant anti-GBM nephritis is an unusual but possibly catastrophic complication of kidney transplantation in AS patients (19).

## Treatment

At present, there is no curative treatment for Alport syndrome. The use of renin-angiotensin-aldosterone system (RAAS) inhibition (RAASi) is the current standard of care to slow progression of kidney disease.

### RAAS Inhibition

Gross and colleagues published in 2012 that use of angiotensin converting enzyme inhibitors (ACEi) in a cohort of 174 patients with AS (predominantly males with XLAS) was associated with a later onset of renal replacement therapy and longer life expectancy compared to a cohort of 109 untreated AS relatives over a mean follow up of more than two decades. This benefit was greater in those patients who started therapy at an earlier stage, especially in those patients who had isolated hematuria or microalbuminuria at the time of treatment initiation (20). Data from the European Alport Registry showed that the use of RAAS blockade in heterozygous Alport carriers is associated with a slower kidney disease progression. Mean age at onset of therapy was 28 years and average time on therapy was 5.8 years on this analysis. Onset of renal replacement therapy (RRT) occurred less frequently and significantly later in patients who were treated with RAAS blockade (21). Yamamura and colleagues examined a group of Japanese male patients with XLAS with proven *COL4A5* variants and showed that the renal protective effect of RAAS blockade was present regardless of the type of *COL4A5* gene variant (truncating vs. non-truncating) (22).

Most ACE Inhibitors are currently authorized by the Food and Drug Administration (FDA) for treatment of hypertension in adults or children ≥6 years old (23). However, the EARLY PROTECT trial showed that use of ramipril in children with AS aged ≥2 years when there is either isolated microscopic hematuria or microalbuminuria (defined as 30–300 mg albumin/g creatinine) is safe and effective at slowing kidney disease progression compared to placebo. Ramipril was initiated at a mean age of 8.8 ± 4.2 years. In this study, ramipril therapy reduced the risk of disease progression by almost 50%. Disease progression was defined as progression to the next disease stage according to the extent of renal damage and loss of function. These stages were defined as (0) microhematuria without microalbuminuria, (I) microalbuminuria [30–300 mg albumin/g creatinine], (II) proteinuria [>300 mg albumin/g creatinine], (III) >25% decline of normal kidney function (CrCl), (IV) end stage kidney disease. A hazard ratio of 0.51 translates to 5.4 children who need to be treated with ramipril for 3 years to prevent one progression of the disease in one child (24).

New clinical practice recommendations support earlier initiation of ACEi in patients affected with AS. Therapy should be started at the time of diagnosis in men with XLAS and in all patients with ARAS, if age ≥24 months. In females with XLAS and in males and females with ADAS, therapy can be started at the onset of microalbuminuria (25).

### Mineralocorticoid Receptor Blockers

Serum aldosterone levels remain elevated in some patients treated with ACEi and/or ARBs, a phenomenon known as “aldosterone breakthrough” which has been correlated with left ventricular hypertrophy and higher rates of albuminuria (26). Rubel and colleagues evaluated if the concomitant use of spironolactone on top of ramipril in *COL4*α*3*^−/−^ mice offered any additional benefit compared to ramipril monotherapy. Dual therapy resulted in a slower kidney disease progression and decreased proteinuria and fibrosis. However, survival did not change. This was possibly due to premature death from side effects of dual therapy such as hyperkalemia (27). Spironolactone effects in human subjects are being evaluated in an observational clinical trial [NCT02378805] (28). The observed favorable outcomes of the non-steroidal mineralocorticoid receptor blocker finerenone on delaying CKD progression in patients with diabetic kidney disease (DKD) (29, 30) suggest the need to test its efficacy in non-diabetic CKD.

## Sodium-Glucose Cotransporter-2 Inhibitors (SGLT2i)

SGLT2i block renal glucose absorption in the proximal convoluted tubule which promotes glucosuria and lowers blood glucose. These drugs have nephroprotective properties which are independent of glycemic control and are thought to be mediated by glucose-induced osmotic diuresis and concomitant natriuresis leading to afferent arteriole vasoconstriction and a reduction in intraglomerular pressure and reduction in albuminuria (31). There is extensive evidence about the benefit of these drugs to slow the progression of CKD of diabetic and non-diabetic origin (32, 33). In a cohort of 5 pediatric patients with Alport syndrome (mean age 10.4 years) with an eGFR >60 ml/min/1.73 m^2^ the use of dapaglifozin was well tolerated and resulted in a 22% reduction in proteinuria by 12 weeks (34).

In diabetic rats, SGLT2i also reduced cardiac accumulation of toxic lipids and free fatty acid uptake (35). In patients with non-alcoholic fatty liver disease and type 2 diabetes mellitus (T2DM) the use of SGLT2i reduced liver fat and improved ALT levels (36). Therefore, it is conceivable that the nephroprotective effect of SGLT2i is also mediated by reducing renal lipotoxicity, which we have found to be pathogenetic in experimental AS (37). While the DAPA-CKD trial suggests a beneficial effect of SGLT2 inhibitors in CKD of non-metabolic origin, the number of patients with Alport syndrome included on this cohort was low and therefore conclusions cannot be extrapolated for this population at this time. However, the efficacy of SGTL2 inhibitors in the treatment of AS should be explored (38).

## New Therapies for Alport Syndrome

AS has become a very appealing disease for drug companies to target for multiple reasons: (i) AS is a great example of progressive CKD with proteinuria and fibrosis that can be a model of other more common causes of kidney disease, (ii) there are no curative therapies for AS, (iii) patients with AS are young and often have no other comorbidities which makes them excellent candidates for clinical trials, (iv) there is a large population affected which would benefit from treatment and (v) any new medication approved for this condition will receive orphan drug designation with might have associated benefits, such as shortened time to approval, monetary benefits and a period of market exclusivity (39). Therefore, the search for a curative treatment for Alport syndrome continues (Table 2).

**Table 2:** Clinical trials for Alport Syndrome listed on clinicaltrials.gov.

**Drug name and sponsor**	**Mechanism of action**	**Status**	**Phase and study name**	**Number of patients**	**Intervention**	**ClinicalTrials.gov Identifier**
Bardoxolone methyl Reata Pharma-ceuticals	Nrf2 activator NF-κB inhibitor	Completed Recruiting	Phase 3 CARDINAL Phase 3 EAGLE	157 480	Oral bardoxolone vs placebo for 100 weeks Oral bardoxolone for up to 5 years (single arm)	NCT03019185 NCT03749447
Lademirsen (RG-012, SAR339375) Genzyme, a Sanofi Company	Anti-microRNA-21 drug. Reduces P42/P44 MAPK pathway activation	Active, not recruiting	Phase 2 HERA	43	Weekly subcutaneous injection of anti-microRNA-21 drug vs. placebo for 110 weeks	NCT02855268
Sparsentan Travere Therapeutics	Dual endothelin type A receptor and angiotensin II type 1 receptor antagonist	Recruiting	Phase 2 EPPIK	57	Oral sparsentan for 108 weeks (single arm) Includes pediatric patients with AS and other proteinuric kidney diseases	NCT05003986
Atrasentan Chinook Therapeutics	Endothelin type A receptor antagonist	Recruiting	Phase 2 AFFINITY	80	Oral atrasentan for 52 weeks (single arm) Includes adult patients with AS and other proteinuric kidney diseases	NCT04573920
Hydroxychloroquine (HCQ) Shanghai Children's Hospital	Immunomodulatory drug	Recruiting	Phase 2 CHXLAS	50	Oral HCQ + benazepril vs. benazepril monotherapy for 6 months	NCT04937907
Benazepril, valsartan and Fluvastatin Mario Negri Institute for pharmacologic research	Drug combination inhibits ACE, ARB and HMG CoA reductase	Completed	Phase 2. Effects of an Intensified treatment with ACE-I, ATA II and statins in AS	9	Drugs: benazepril, valsartan and Fluvastatin (single arm)	NCT00309257
Ramipril Institut fuer anwendumgsorientierte Forschung und klinische Studien GmbH	Angiotensin converting enzyme inhibitor	Completed	Phase 3. Early prospective therapy trial to delay renal failure in Children with AS (EARLY PROTECT)	66	Ramipril (blinded) Placebo to ramipril Ramipril (open-label)	NCT01485978
R3R01 & River 3 Renal Corp	Lipid-modifying drug	Not yet recruiting	Phase 2. Study to evaluate R3R01 in patients with Alport syndrome and patients with Focal Segmental Glomerulosclerosis	50	Oral R3R01 for 12 weeks	NCT05267262

### Bardoxolone

Bardoxolone methyl is a semisynthetic triterpenoid that activates nuclear factor erythroid 2-related factor 2 (Nrf2), a transcription factor that modulates the expression of multiple genes involved in inflammation, oxidative stress, and cellular energy metabolism (40, 41). By activating the Keap1-Nrf2 pathway, bardoxolone methyl also reduces nuclear factor kappa-light-chain-enhancer of activated B-cells (NF-κB), the primary transcription factor producing proteins causative of inflammation and the generation of reactive oxygen species (42, 43). Bardoxolone increased GFR in patients with T2DM and CKD stage 3 over a 52-week period (44) which led to the creation of the BEACON trial, a randomized clinical trial (RCT) that evaluated the efficacy and safety of bardoxolone in patients with T2DM and CKD stage 4. Unfortunately, there was a high number of heart failure hospitalizations in the patients treated with bardoxolone which resulted in early trial discontinuation. There was also an increase in blood pressure and an increase in proteinuria in the bardoxolone group (45).

More recently, the CARDINAL phase 3 study evaluated the use of bardoxolone methyl in a group of patients with AS. 157 patients were randomly assigned to receive once-daily oral bardoxolone (*n* = 77) or placebo (*n* = 80). The primary endpoint was the change in eGFR after 100 weeks of treatment compared to baseline. Average age at screening was 39.2 years. The mean baseline eGFR was 62.7 ml/min/1.73 m^2^ and mean UACR was 141 mg/g (46).

The FDA has analyzed data from CARDINAL and has concluded that there is no evidence that bardoxolone is effective at slowing kidney disease progression in patients with AS. The FDA also raised major efficacy and safety concerns related to bardoxolone use. It has been speculated that bardoxolone leads to an increase in intraglomerular pressure, which over time could have detrimental effects that might lead to an accelerated progression to kidney failure. It has also been suggested that the 4-week washout period was not long enough to resolve the reversible pharmacodynamic effect of bardoxolone and that the time to resolution of such effect is predicted to be at least 60 days (based on an elimination half-life of 15 days of bardoxolone). Major concerns regarding drug safety are worsening albuminuria, hypertension and a decrease in body weight. There is a lack of data using bardoxolone in animal models of Alport syndrome or other adequately controlled clinical trials in AS or CKD to show that bardoxolone is effective at delaying kidney function decline.

### Anti-microRNA-21

MicroRNAs (miRNAs) are short non-coding RNAs which can regulate gene expression by inhibiting the translation or increasing the degradation of their target messenger RNAs (47). The ability of a single miRNA to regulate multiple downstream target mRNAs altered in disease conditions makes miRNAs attractive therapeutic targets with the potential to impact a variety of molecular pathways (48). miRNA-21 has been found to be dysregulated in multiple kidney disorders, including AS. It was shown that renal miRNA-21 is upregulated in *Col4*α*3*^−/−^ mice and the use of anti-miRNA-21 oligonucleotides significantly slows kidney disease progression and improves survival in Alport mice (49). In patients with AS, the expression of miRNA-21 in kidney samples was found to be significantly higher compared to controls and it was correlated with severity of kidney disease as evidenced by proteinuria, kidney function biomarkers and renal pathology scores (50). A phase 2 RCT of lademirsen (previously known as RG-012) which is a subcutaneous injection of an anti-miRNA-21 molecule is underway (HERA clinical trial [NCT02855268]). This trial, sponsored by Sanofi, will enroll a total of 45 patients with AS and results are anticipated to be accesible by 2023 (51).

### Endothelin Type A Receptor (ETAR) and Angiotensin II Type 1 Receptor (AT1R) Inhibitors

The activation of ETAR has an important role in renal and inner ear pathologies in patients with AS. Despite being standard of care in patients with AS, the use of RAASi does not mitigate the impact on hearing.

Sparsentan, a dual ETAR/AT1R inhibitor, was able to extend lifespan in AS mice and lead to greater reductions in proteinuria compared to a selective AT1R inhibitor (losartan) or selective ETAR inhibitor (atrasentan) when treatment was initiated at 4 weeks. Preventive use of sparsentan was also able to mitigate the structural and functional auditory changes in AS mice. This auditory benefit was not observed with losartan (52).

The clinical trial EPPIK (NCT05003986) is evaluating use of sparsentan oral suspension in a pediatric population with AS with UPCR ≥1.0 g/g and other proteinuric glomerular diseases (53).

Sparsentan showed greater reduction in proteinuria compared to irbesartan after 8 weeks of treatment in patients with FSGS in a phase 2 RCT. In the patients treated with sparsentan, 16.4% of patients developed hypotension and 12.3% reported edema, but none of these adverse events were serious and there were no patients who dropped out from the study (54). The phase 3 DUPLEX study is ongoing and this will evaluate extended antiproteinuric efficacy and kidney protective potential of dual ETAR and AT1R blockade in patients with FSGS (55).

Atrasentan is an oral selective ETAR inhibitor that reduced albuminuria and risk of renal events in patients with diabetes and CKD. Edema and anemia were more frequent in the atrasentan group compared to placebo (56, 57).

AFFINITY (NCT04573920) is a phase 2 open-label trial of atrasentan which is currently recruiting patients and it will include a cohort of 80 patients with AS as well as other proteinuric kidney diseases (58).

### Lipid-Modifying Drugs

The frequency of cardiovascular disease (CVD) is much higher in patients with CKD compared to those without CKD. The majority of deaths in patients with CKD are attributed to CVD, mainly atherosclerotic heart disease, cardiac arrhythmias and cardiac arrest. Individuals with familial hypercholesterolemia are at increased risk of developing CKD (59). Irrespectively of systemic hyperlipidemia accompanying CKD, research from us and others has shown that renal lipotoxicity rather than systemic dyslipidemia is the main contributor to the pathogenesis and progression of proteinuric kidney diseases (60–67). The accumulation of excessive lipids in non-adipose tissue is described as lipotoxicity and this is associated with cell dysfunction and apoptosis (68). We have recently described an important accumulation of triglycerides (69) and esterified cholesterol (37, 70, 71) in the kidney cortex of experimental AS. In fact, lipid droplets, foamy podocytes and foamy interstitial cells are frequently seen in kidney biopsies of patients with AS, but their origin remains unknown.

An evolving research field is identifying key enzymes or transporters in intrarenal lipid homeostasis as potential therapeutic targets to treat proteinuric kidney diseases.

When there is cholesterol deficit, cholesterol can be synthesized *de novo via* 3-hydroxy-3-methylglutaryl-CoA reductase (HMGCR) or cholesterol attached to low-density lipoprotein (LDL) can be released into the cells through the LDL receptor (LDLR). Conversely, excessive cholesterol can be extracted from the cell through ATP-binding cassette A1 (ABCA1) and ATP-binding cassette subfamily G member 1 (ABCG1). Accumulation of cellular cholesterol stimulates the cholesterol-esterifying enzyme sterol O-acyltransferase (SOAT1), which transforms free cholesterol to cholesterol esters that are deposited in lipid droplets. In podocytes, the uptake of free fatty acids for triglyceride synthesis occurs through cluster of differentiation 36 (CD36) (65).

Excessive aggregation of free cholesterol results in cellular toxicity. Maintenance of adequate levels of free cholesterol is expedited by cholesterol esterification and reverse cholesterol transport (RCT) pathways which induce the efflux of cholesterol to HDL. Impaired RCT can promote glomerulosclerosis and tubulointerstitial damage (72). Glomerular and tubular lipid accumulation has been described in experimental models of kidney disease of metabolic and non-metabolic origin, including DKD (61, 63, 73), FSGS and AS (69–71, 74) and this is attributable to abnormalities in lipid metabolism. Among different lipid species, esterified cholesterol accumulation in kidney cortex correlates best with kidney outcomes compared to accumulation of other lipids (75).

### Statins and Ezetimibe

The use of statins in CKD patients is essential to reduce cardiovascular risk. KDIGO 2013 guidelines recommend use of a statin in adults aged ≥50 years with eGFR <60 ml/min/1.73m^2^. This does not apply for end stage kidney disease patients (1A) (76). It has been speculated that statin treatment may be associated with slower kidney disease progression (77). However, there is no convincing evidence for such statement. The beneficial effects of statins observed in *Col4a3*^−/−^ mice (78) have not been confirmed in patients with AS. The use of ezetimibe was found to partially protect from albuminuria and from CKD progression in *Col4a3*-/- mice in association with reduced triglyceride content. In these studies, ezetimibe was found to reduce CD36 dependent fatty acid uptake (69). Whether this effect may be observed in patients affected with AS remains to be established.

### Inducers of Cholesterol Efflux

The expression of cholesterol efflux transporter ABCA1 is reduced in glomeruli of individuals with T2DKD (61) as well as in experimental models of AS and FSGS (62, 70). There is a causative directional link between TNF expression in glomerular cells and an altered cholesterol efflux *via* ABCA1 and decreased cholesterol esterification by SOAT1 resulting in cholesterol aggregation and increased risk of proteinuria (71, 75). Studies have shown that either genetic or drug-induced activation of ABCA1 or use of a cholesterol sequestrant is effective in reducing proteinuria in AS, DKD and FSGS.

We previously proved that in the *COL4*α*3* knockout (KO) mice (AS mice) there is glomerular accumulation of cholesterol esters which suggests that CKD in AS may be a disorder of fatty kidney disease. Administration of hydroxypropyl-β-cyclodextrin (HPβCD), a cholesterol-chelating sugar, was found to protect AS mice from developing proteinuria, progressive kidney disease and renal fibrosis and this was associated with a lower concentration of kidney cholesterol esters, lipid droplets and cholesterol crystals, but also with increased serum HDL levels. Moreover, HPβCD treatment resulted in prolonged lifespan (70).

Unfortunately, the cholesterol removal effect of cyclodextrin is not selective, and its parenteral route of administration is impractical. Concerns have been raised about potential ototoxicity of HPβCD due to loss of cochlear outer hair cells which would be of particular concern in patients with AS who are already susceptible to hearing loss (79).

More recently, we developed a series of small molecule oral agents belonging to the class of 5-arylnicotinamide compounds (Cpds). These Cpds increase ABCA1-dependent cholesterol efflux by targeting Oxysterol Binding Protein Like 7 (OSBPL7). OSBPL7 is present in the kidney, including the glomeruli and podocytes. Cpds were able to induce ABCA1 and cholesterol efflux from cholesterol-repleted podocytes *in vitro* and decreased proteinuria, attenuated kidney function decline and prolonged lifespan in mouse models of AS and FSGS, even when the Cpds were administered in mice with established CKD and proteinuria. Despite reducing the amount of cholesterol esters in the kidney in the *COL4*α*3* KO mice the serum cholesterol levels did not change, which suggests that these Cpds may be protective mainly by reducing the concentration of cholesterol and lipids in target organs (37). These Cpds have a greater antiproteinuric effect compared to the one of ramipril previously reported in a syngeneic model of AS (69). Unlike the Cpds, the administration of RAAS blockade did not prolong lifespan in older *COL4*α*3* mice, despite being the most effective therapy for early intervention in this model (80–82). An oral molecule that increases the functional activity of the ABCA1 transporter is currently under development. However, this information is not publicly available at this time.

### Discoidin Domain Receptor 1 (DRR1) Inhibitors

DDR1 is a receptor tyrosine kinase that is stimulated by collagens. It is involved in the development of fibrotic disorders. DDR1 deficient *Col4a3*-/- mice were reported to be protected from proteinuria and renal failure, suggesting DDR1 as an amenable therapeutic target (82). Pharmacological inhibition of DDR1 in *COL4*α3^−/−^ mice caused mild reductions in albuminuria and renal fibrosis (83). More recently, we have described a new mechanism linking DDR1 activation to renal lipotoxicity. *De novo* production of the collagen type 1 has been observed in *Col4*α*3* knockout mice (AS mice). Collagen 1 activates DDR1 and induces CD36-mediated podocyte lipotoxic injury and disruption of podocyte-GBM interaction with subsequent development of proteinuria. CD36 induces oxidative stress and apoptosis in podocytes by increasing intake of free fatty acids and accumulation of intracellular triglycerides. Therefore, the clinical development of DDR1 inhibitors for the treatment of AS remains an interesting opportunity.

### Osteopontin Blocking Agents

Osteopontin (OPN) is a protein with an important role as a regulator of inflammation, heart failure and tumor metastases (84, 85). In animal models of albuminuria, kidney OPN mRNA and protein levels were elevated and in children with nephrotic syndrome, urinary OPN levels were elevated (86). Ding and colleagues demonstrated that OPN expression is increased in the renal tubules of the *Col4*α*3*^−/−^ AS mouse where it regulates dynamin-3-mediated (DNM3) increased LDL receptor (LDLR) expression, increases LDL-cholesterol influx and leads to defective mitochondrial bioenergetics.

OPN genetic deletion caused a significant reduction in the expression of DNM3 and LDLR in the AS mouse and reduced albuminuria, tubulointerstitial inflammation, apoptosis, hypertension, hearing loss and visual deficits (74). OPN blocking agents such as antibody or RNA aptamer might be a potential therapeutic target in patients with AS.

### Hydroxychloroquine

The antimalarial drug hydroxychloroquine (HCQ) is an immunomodulatory drug commonly used to treat rheumatologic diseases such as systemic lupus erythematosus and rheumatoid arthritis. It blocks the immune system by inhibiting Toll-like receptor signaling and suppressing cytokine production and T cells by decreasing the expression of CD154 (87).

A phase 2 RCT of patients with IgA nephropathy (IgAN) who were receiving optimized RAAS inhibitor therapy and had a mean eGFR 54 ml/min/1.73 m^2^ and median proteinuria of 1.7 g/d showed that the addition of HCQ compared to placebo significantly reduced proteinuria at 6 months (0.9 vs. 1.9 g/d; P 0.002) (88). A case control study compared the safety and efficacy of HCQ and glucocorticoids in individuals with IgAN with proteinuria >1 g/d despite optimization of RAASi treatment. The reduction in proteinuria achieved by HCQ was only slightly lower to glucocorticoids over a period of 6 months. However, HCQ was safer than glucocorticoid treatment (89).

A phase 2 RCT to assess the safety and effectiveness of HCQ in patients with X-linked Alport syndrome (NCT04937907) is under way in China. Enrollment includes AS patients who have been on a stable dose of enalapril for 6 months (90).

### Metformin

Metformin is an oral hypoglycemic agent which effectively reduces HbA1c in patients with T2DM with a low risk for hypoglycemia. In a mouse model of AS, use of metformin reduced proteinuria and ameliorated progression of kidney disease. Metformin suppressed renal inflammation, fibrosis and glomerular injury. The dual administration of metformin and losartan prolonged survival of AS mice when compared to losartan monotherapy. Both metformin and losartan modified podocyte-related molecular pathways associated to inflammation and metabolism according to transcriptome analysis. In addition, metformin was found to regulate multiple genes also related to metabolism which were not affected by losartan (91). This study raises the possibility that combination of RAASi and metformin may slow CKD progression and prolong survival also in non-diabetic chronic kidney disease. Considering the low cost and wide availability of metformin this might be explored further in research trials. It is important to mention that metformin is excreted unchanged in the urine since it is not metabolized and the FDA applied a box warning to this drug and recommends to avoid its use in advanced kidney disease (eGFR <30 ml/min/1.73 m^2^) due to its potential association with lactic acidosis.

### Paricalcitol

Paricalcitol is an active vitamin D analog. An elevated parathyroid hormone (PTH) and a low 1,25-dihydroxyvitamin D are associated with lower bone density, heart disease, blunted immune system and higher mortality in patients with ESKD. Recent studies in hemodialysis patients suggest that paricalcitol is associated with a better survival and tolerability compared to calcitriol (92). It is possible that activation of vitamin D receptors has beneficial effects on the cardiovascular system that reduce mortality in CKD and that this is independent of calcium, phosphorus and PTH. Paricalcitol suppresses renin production at higher than conventional doses (93, 94). Downregulation of the RAAS may be the mechanism by which paricalcitol has some nephroprotective and antifibrotic effects.

### Chaperones

Protein misfolding in the endoplasmic reticulum (ER) leads to loss of function or toxic effect designated as ER stress in genetic diseases caused by genetic mutations. Inappropriately folded proteins are generally recognized by the cell quality-control mechanisms and are retained or destroyed in the ER. Chaperones are ubiquitous molecules that enable correct protein assembly by attaching to and stabilizing unfolded proteins allowing them to become functional and correctly routed to the location where they will reside.

Use of chaperone therapies as a potential therapeutic approach to protein misfolding diseases has become one of the major targets of clinical research. Migalastat is an oral chaperone which stabilizes mutant α-galactosidase and facilitates adequate enzyme routing to lysosomes and is used to treat patients affected with Fabry's disease (95). Chaperones are useless when there is absence of protein which is the case in diseases caused by truncating mutations (deletions, frameshift and nonsense mutations). However, estimates show that around 40–50% of males with XLAS (96, 97) and around 40% in male and female patients with ARAS (98) exhibit missense mutations as the cause of their AS.

A study in fibroblast cell lines of men with XLAS showed that the use of chaperone sodium 4-phenyl butyric acid (PBA) increased levels of collagen IV α5 mRNA and decreased ER stress and autophagy (99). When oral treatment with the chaperone PBA was administered to *COL4*α*1* mutant mice, either as a preventative treatment or for those with established disease, there were fewer intracranial hemorrhage cases. However, PBA did not prevent from developing eye and kidney defects (100).

### Genome Editing Therapy

Genome editing therapy is an experimental technique that aims to correct defective genes in order to cure a disease. It can be executed *via* different methodologies which include rendering deleterious mutations inactive, adding protective mutations or therapeutic transgenes, or distortion of viral DNA (101, 102). Replacement of a mutant allele by a corrected copy of the gene requires the effective delivery of the latter *via* a vehicle, for instance a virus or nanoparticle, to an approachable tissue compartment (103). If the normal gene substitutes the abnormal allele there will be proliferation of these new modified cells which may generate the desired protein in sufficient amounts to restore a normal phenotype (104). Here we describe some of the therapeutic concepts currently under investigation.

The Clustered Regularly-Interspaced-Short Palindromic Repeat (CRISPR/Cas9) system has became a promising gene editing therapy for many rare genetic disorders, with successful *in vitro* results in cases of homozygous β-thalassemia generating functional red blood cells precursors (102), but with less success in the manipulation of podocytes and their regeneration.

The CRISPR/Cas9 system consists of two components: a single-guide RNA (sgRNA) and an endonuclease Cas9. The sgRNA can guide Cas9 to the genomic site of action where a highly precise double strand break (DSB) should occur following a Watson-Crick base pairing recognition. DSBs caused by CRISPR/Cas9 are generally corrected through non-homologous end joining which may generate deletions and insertions. However, the precision of the DSB correction can be augmented by using a “repair template” donor DNA compatible with the genomic region of interest which can be used to synthesize de-novo wild type DNA (105).

A recent study by Daga et al. (106), demonstrated that it is feasible to obtain podocyte-lineage cells derived from urine recreating the physiological conditions seen in these particular cells, allowing to effectively define the *COL4* variants correction index after experimental interventions. This novel tool was applied for two different forms of hereditary transmission in AS, X-linked and autosomal dominant variants, using a self-inactivating dual-plasmid approach where a homologous repair is mainly induced by a self-cleaving streptococcus pyogenes Cas9 (SpCas9) coupled with a sgRNA from CMV inducing DNA damage, while the other plasmid carries a double stranded DNA (dsDNA) donor fragment. This approach resulted in very high correction rates which ranged from 44% in the *COL4A3* gene to 58% in the *COL4A5* gene and it led to a reduced percentage of indels (10.4% for *COL4A3* and 8.8% for *COL4A5*).

The number of podocytes decreases significantly over time in patients with AS as a consequence of structural defects which lead to apoptosis and cell death. CRISPR/Cas9 gene therapy is expected to be more effective in the early phase of the disease since at this time a functional GBM can still be potentially restored (106). Despite these promising results, there is a long way ahead of us until this proof of concept can be transitioned to *in vivo* experiments due to the difficulty in manipulation of podocytes.

Lin et al. used a *COL*4*A*3^−^/^−^ mouse model of AS and an inducible transgene system and they discovered that the podocyte secretion of α3α4α5(IV) heterotrimers into a defective GBM was effective at restoring the absent collagen IV network which slowed the course of renal disease and prolonged survival (107). Funk et al. increased the expression of *COL4*α*3* transgene in endothelial cells of *COL*4*A*3^−^/^−^ Alport mice through adenovirus-mediated gene transfer. Unfortunately, these AS mice did not exhibit *COL4A3/A4* or assembled α3α4α5(IV) heterotrimer staining and subsequent resolution of the disease specific phenotype was not achieved (108).

Reports of succesful delivery of *COL4A5* gene into swine kidney with an adenovirus vector resulted in increased deposition of α5 (IV) collagen into the GBM. However, this technique is impractical since it requires to administer the vector directly in the renal artery which may not be possible in human trials (109).

Another concept being explored in AS is the X-chromosome (Xc) reactivation approach. It is known that during the early embryological development of females, an Xc on each cell is randomly inactivated (lyonization), ensuring an even expression of cells of female XX compared to male XY (110). Based on this premise, the idea of reactivating the healthy copy of *COL4A5* gene on the inactivated Xc has been contemplated in cases of X-linked AS in women. Unfortunately, this concept continues to be in the early experimental phase, with the major limiting factor being concerns regarding off-target effects.

### Stem Cell Therapy

The use of induced pluripotent stem cell (iPSC) lines is a valuable tool to study AS pathologic mechanisms and evaluate the efficacy of experimental therapies. The rationale of the potential use of iPSCs as a therapy in AS is based on the concept that these cells will eventually migrate and engraft into the renal glomeruli, differentiating into functional podocytes and generating a new healthy GBM (111). One study proved the efficacy of stem cell transplantation of wild-type bone marrow into irradiated *COL*4*A*3^−^/^−^ mice which partially restored the expression of the type IV collagen α3 chain, reducing proteinuria and overall improving kidney histology (112). Another study demonstrated that transplanting first trimester human fetal chorionic stem cells into a mouse model of AS delays kidney disease progression (113). Despite the promising results, other studies have failed to demonstrate the same effect in cases of *COL4A3* deficient mice (114). Bone marrow transplantation is considered as a last resource measure for life threatening diseases, given its high mortality risk along with the uncertainty of the available studies using this method, hence is not considered as a potential treatment of AS.

## Discussion

Alport syndrome is a frequent inherited kidney disease for which currently there is no curative treatment available. Conventional treatment aims to slow progression to kidney failure using RAASi. Drug development in AS is currently under way with drug targets at different stages of the disease. Experimental drugs may target the altered function of the GBM or the interaction between GBM with podocytes and endothelial cells. Other new drugs target tubular cell injury or the inflammation and fibrosis seen in late stages of the disease. Targeting defective collagen chains early in the disease through gene therapy might be the therapeutic approach with the highest potential to reverse this disorder. Research advances on the innovative therapeutic approaches for this condition are exciting and may bring hope to the millions of patients affected.

## Author Contributions

EC prepared manuscript draft and created the tables. JR prepared manuscript draft. YD and AF revised the manuscript. All authors contributed to the article, reviewed, and approved the final version of the manuscript.

## Funding

Research in AF laboratory is supported by the National Institute of Health [Grant Numbers R01DK117599, R01DK104753, R01CA227493, U54DK083912, UM1DK100846, and U01DK116101] and by the Miami Clinical Translational Science Institute [Grant Number UL1TR000460]. He also received support from the Alport Syndrome Foundation, Boehringer Ingelheim, and Hoffman La Roche. YD is supported by the KL2 program of the Miami Clinical Translational Science Institute [Grant Number UL1TR000460].

## Conflict of Interest

AF is Chief Scientific Officer of L&F Health LLC, holds equity interests in L&F Research and is the inventor of assets developed by ZyVersa Therapeutics. ZyVersa has licensed worldwide rights to develop and commercialize hydroxypropyl-beta-cyclodextrin for treatment of kidney disease from L&F Research. AF is the scientific founder and holds equity in River 3 Renal Corporation. The remaining authors declare that the research was conducted in the absence of any commercial or financial relationships that could be construed as a potential conflict of interest.

## Publisher's Note

All claims expressed in this article are solely those of the authors and do not necessarily represent those of their affiliated organizations, or those of the publisher, the editors and the reviewers. Any product that may be evaluated in this article, or claim that may be made by its manufacturer, is not guaranteed or endorsed by the publisher.

## References

[1] Warady BA Agarwal R Bangalore S Chapman A Levin A Stenvinkel P. Alport syndrome classification and management. Kidney Med. (2020) 2:639–49. 10.1016/j.xkme.2020.05.014 33094278 PMC7568086

[2] Suh JH Miner JH. The glomerular basement membrane as a barrier to albumin. Nat Rev Nephrol. (2013) 9:470–7. 10.1038/nrneph.2013.109 23774818 PMC3839671

[3] Kashtan CE Ding J Garosi G Heidet L Massella L Nakanishi K. Alport syndrome: a unified classification of genetic disorders of collagen iv α345: a position paper of the alport syndrome classification working group. Kidney Int. (2018) 93:1045–51. 10.1016/j.kint.2017.12.018 29551517

[4] Adam J Connor TM Wood K Lewis D Naik R Gale DP. Genetic testing can resolve diagnostic confusion in Alport syndrome. Clin Kidney J. (2014) 7:197–200. 10.1093/ckj/sft144 24944784 PMC3970340

[5] Savige J. Should we diagnose autosomal dominant alport syndrome when there is a pathogenic heterozygous *COL4A3* or *COL4A4* Variant?. Kidney Int Rep. (2018) 3:1239–41. 10.1016/j.ekir.2018.08.002 30450445 PMC6224634

[6] Savige J Lipska-Zietkiewicz BS Watson E Hertz JM Deltas C Mari F. Guidelines for genetic testing and management of alport syndrome. Clin J Am Soc Nephrol. (2021) 20:CJN.04230321. 10.2215/CJN.04230321 34930753 PMC8763160

[7] Gibson J Fieldhouse R Chan MMY Sadeghi-Alavijeh O Burnett L Izzi V. Genomics england research consortium. prevalence estimates of predicted pathogenic *COL4A3-COL4A5* variants in a population sequencing database and their implications for alport syndrome. J Am Soc Nephrol. (2021). 32:2273–90. 10.1681/ASN.2020071065 34400539 PMC8729840

[8] Hays T Groopman EE Gharavi AG. Genetic testing for kidney disease of unknown etiology. Kidney Int. (2020) 98:590–600. 10.1016/j.kint.2020.03.031 32739203 PMC7784921

[9] Fogo AB Lusco MA Najafian B Alpers CE. AJKD atlas of renal pathology: alport syndrome. Am J Kidney Dis. (2016) 68:e15–6. 10.1053/j.ajkd.2016.08.002 27664477

[10] Jais JP Knebelmann B Giatras I De Marchi M Rizzoni G Renieri A. X-linked Alport syndrome: natural history and genotype-phenotype correlations in girls and women belonging to 195 families: a “European community alport syndrome concerted action” study. J Am Soc Nephrol. (2003) 14:2603–10. 10.1097/01.ASN.0000090034.71205.74 14514738

[11] Kashtan CE Segal Y Flinter F Makanjuola D Gan JS Watnick T. Aortic abnormalities in males with Alport syndrome. Nephrol Dial Transplant. (2010) 25:3554–60. 10.1093/ndt/gfq271 20494893 PMC2980995

[12] Storey H Savige J Sivakumar V Abbs S Flinter FA. COL4A3/COL4A4 mutations and features in individuals with autosomal recessive Alport syndrome. J Am Soc Nephrol. (2013) 24:1945–54. 10.1681/ASN.2012100985 24052634 PMC3839543

[13] Furlano M Martínez V Pybus M Arce Y Crespí J Venegas MDP. Clinical and genetic features of autosomal dominant alport syndrome: a cohort study. Am J Kidney Dis. (2021) 78:560–70.e1. 10.1053/j.ajkd.2021.02.326 33838161

[14] Gast C Pengelly RJ Lyon M Bunyan DJ Seaby EG Graham N. Collagen (COL4A) mutations are the most frequent mutations underlying adult focal segmental glomerulosclerosis. Nephrol Dial Transplant. (2016) 31:961–70. 10.1093/ndt/gfv325 26346198

[15] Yao T Udwan K John R Rana A Haghighi A Xu L. Integration of genetic testing and pathology for the diagnosis of adults with FSGS. Clin J Am Soc Nephrol. (2019) 14:213–23. 10.2215/CJN.08750718 30647093 PMC6390925

[16] Petzold F Bachmann A Bergmann C Helmchen U Halbritter J. Retrospective genetic analysis illustrates the spectrum of autosomal Alport syndrome in a case of living-related donor kidney transplantation. BMC Nephrol. (2019) 20:340. 10.1186/s12882-019-1523-7 31477057 PMC6721183

[17] Gross O Weber M Fries JW Müller GA. Living donor kidney transplantation from relatives with mild urinary abnormalities in Alport syndrome: long-term risk, benefit and outcome. Nephrol Dial Transplant. (2009) 24:1626–30. 10.1093/ndt/gfn635 19028755

[18] Choi C Ahn S Min SK Ha J Ahn C Kim Y. Midterm outcome of kidney transplantation from donors with thin basement membrane nephropathy. Transplantation. (2018) 102:e180–e4. 10.1097/TP.0000000000002089 29334529

[19] Kashtan CE. Renal transplantation in patients with Alport syndrome: patient selection, outcomes, and donor evaluation. Int J Nephrol Renovasc Dis. (2018) 11:267–270. 10.2147/IJNRD.S150539 30410383 PMC6198874

[20] Gross O Licht C Anders HJ Hoppe B Beck B Tönshoff B. Early angiotensin-converting enzyme inhibition in Alport syndrome delays renal failure and improves life expectancy. Kidney Int. (2012) 81:494–501. 10.1038/ki.2011.407 22166847

[21] Temme J Peters F Lange K Pirson Y Heidet L Torra R. Incidence of renal failure and nephroprotection by RAAS inhibition in heterozygous carriers of X-chromosomal and autosomal recessive Alport mutations. Kidney Int. (2012) 81:779–83. 10.1038/ki.2011.452 22237748

[22] Yamamura T Horinouchi T Nagano C Omori T Sakakibara N Aoto Y. Genotype-phenotype correlations influence the response to angiotensin-targeting drugs in Japanese patients with male X-linked Alport syndrome. Kidney Int. (2020) 98:1605–14. 10.1016/j.kint.2020.06.038 32712167

[23] Chu PY Campbell MJ Miller SG Hill KD. Anti-hypertensive drugs in children and adolescents. World J Cardiol. (2014) 6:234–44. 10.4330/wjc.v6.i5.234 24944754 PMC4062129

[24] Gross O Tönshoff B Weber LT Pape L Latta K. A multicenter, randomized, placebo-controlled, double-blind phase 3 trial with open-arm comparison indicates safety and efficacy of nephroprotective therapy with ramipril in children with Alport's syndrome. Kidney Int. (2020) 97:1275–86. 10.1016/j.kint.2019.12.015 32299679

[25] Kashtan CE Gross O. Clinical practice recommendations for the diagnosis and management of Alport syndrome in children, adolescents, and young adults-an update for (2020). Pediatr Nephrol. (2021) 36:711–9. 10.1007/s00467-020-04819-6 33159213

[26] Bomback AS Klemmer PJ. The incidence and implications of aldosterone breakthrough. Nat Clin Pract Nephrol. (2007) 3:486–92. 10.1038/ncpneph0575 17717561

[27] Rubel D Zhang Y Sowa N Girgert R Gross O. Organoprotective effects of spironolactone on top of ramipril therapy in a mouse model for alport syndrome. J Clin Med. (2021) 10:2958. 10.3390/jcm10132958 34209341 PMC8268845

[28] European Alport Therapy Registry. European initiative towards delaying renal failure in Alport syndrome. Bethesda, MD: National Library of Medicine (2015). Available online at: https://clinicaltrials.gov/ct2/show/NCT02378805

[29] Bakris GL Agarwal R Anker SD Pitt B Ruilope LM. Effect of Finerenone on Chronic Kidney Disease Outcomes in Type 2 Diabetes. N Engl J Med. (2020). 383:2219-29. 10.1056/NEJMoa2025845 33264825

[30] Pitt B Filippatos G Agarwal R Anker SD Bakris GL Rossing P. Cardiovascular Events with Finerenone in Kidney Disease and Type 2 Diabetes. N Engl J Med. (2021) 385:2252–63. 10.1056/NEJMoa2110956 34449181

[31] Vallon V. The mechanisms and therapeutic potential of SGLT2 inhibitors in diabetes mellitus. Annu Rev Med. (2015) 66:255–70. 10.1146/annurev-med-051013-110046 25341005

[32] Perkovic V Jardine MJ Neal B Bompoint S Heerspink HJL Charytan DM. Canagliflozin and renal outcomes in Type 2 diabetes and nephropathy. N Engl J Med. (2019) 380:2295–306. 10.1056/NEJMoa1811744 30990260

[33] Heerspink HJL Stefánsson BV Correa-Rotter R Chertow GM Greene T Hou FF. Dapagliflozin in patients with chronic kidney disease. N Engl J Med. (2020). 383:1436–46. 10.1056/NEJMoa2024816 32970396

[34] Liu J Cui J Fang X Chen J Yan W Shen Q. Efficacy and safety of dapagliflozin in children with inherited proteinuric kidney disease: a pilot study. Kidney Int Rep. (2021) 7:638–41. 10.1016/j.ekir.2021.12.019 35257077 PMC8897303

[35] Aragón-Herrera A Feijóo-Bandín S Otero Santiago M Barral L Campos-Toimil M Gil-Longo J. Empagliflozin reduces the levels of CD36 and cardiotoxic lipids while improving autophagy in the hearts of Zucker diabetic fatty rats. Biochem Pharmacol. (2019) 170:113677. 10.1016/j.bcp.2019.113677 31647926

[36] Kuchay MS Krishan S Mishra SK Farooqui KJ Singh MK Wasir JS. Effect of empagliflozin on liver fat in patients with type 2 diabetes and nonalcoholic fatty liver disease: a randomized controlled trial (E-LIFT Trial). Diabetes Care. (2018) 41:1801–8. 10.2337/dc18-0165 29895557

[37] Wright MB Varona Santos J Kemmer C Maugeais C Carralot JP Roever S. Compounds targeting OSBPL7 increase ABCA1-dependent cholesterol efflux preserving kidney function in two models of kidney disease. Nat Commun. (2021) 12:4662. 10.1038/s41467-021-24890-3 34341345 PMC8329197

[38] Mabillard H Sayer JA. SGLT2 inhibitors - a potential treatment for Alport syndrome. Clin Sci (Lond). (2020) 134:379–88. 10.1042/CS20191276 32064497

[39] Torra R Furlano M. New therapeutic options for Alport syndrome. Nephrol Dial Transplant. (2019) 34:1272–9. 10.1093/ndt/gfz131 31190059

[40] Wardyn JD Ponsford AH Sanderson CM. Dissecting molecular cross-talk between Nrf2 and NF-κB response pathways. Biochem Soc Trans. (2015) 43:621–6. 10.1042/BST20150014 26551702 PMC4613495

[41] Kobayashi EH Suzuki T Funayama R Nagashima T Hayashi M Sekine H. Nrf2 suppresses macrophage inflammatory response by blocking proinflammatory cytokine transcription. Nat Commun. (2016) 7:11624. 10.1038/ncomms11624 27211851 PMC4879264

[42] Wong ET Tergaonkar V. Roles of NF-kappaB in health and disease: mechanisms and therapeutic potential. Clin Sci (Lond). (2009) 116:451–65. 10.1042/CS20080502 19200055

[43] Baker RG Hayden MS Ghosh S. NF-κB, inflammation, and metabolic disease. Cell Metab. (2011) 13:11-22. 10.1016/j.cmet.2010.12.008 21195345 PMC3040418

[44] Pergola PE Raskin P Toto RD Meyer CJ Huff JW Grossman EB. Bardoxolone methyl and kidney function in CKD with type 2 diabetes. N Engl J Med. (2011) 365:327–36. 10.1056/NEJMoa1105351 21699484

[45] de Zeeuw D Akizawa T Audhya P Bakris GL Chin M Christ-Schmidt H. Bardoxolone methyl in type 2 diabetes and stage 4 chronic kidney disease. N Engl J Med. (2013) 369:2492-503. 10.1056/NEJMoa1306033 24206459 PMC4496027

[46] Chertow GM Appel G Andreoli S Bangalore S Block G Chapman A. study design and baseline characteristics of the cardinal trial: a phase 3 study of bardoxolone methyl in patients with alport syndrome. Am J Nephrol. (2021) 52:180–9. 10.1159/000513777 33789284 PMC8220919

[47] Christopher AF Kaur RP Kaur G Kaur A Gupta V Bansal P. MicroRNA therapeutics: Discovering novel targets and developing specific therapy. Perspect Clin Res. (2016) 7:68–74. 10.4103/2229-3485.179431 27141472 PMC4840794

[48] Rupaimoole R Slack FJ. MicroRNA therapeutics: towards a new era for the management of cancer and other diseases. Nat Rev Drug Discov. (2017) 16:203–22. 10.1038/nrd.2016.246 28209991

[49] Gomez IG MacKenna DA Johnson BG Kaimal V Roach AM Ren S. Anti-microRNA-21 oligonucleotides prevent Alport nephropathy progression by stimulating metabolic pathways. J Clin Invest. (2015) 125:141–56. 10.1172/JCI75852 25415439 PMC4382246

[50] Guo J Song W Boulanger J Xu EY Wang F Zhang Y. Dysregulated Expression of microRNA-21 and disease-related genes in human patients and in a mouse model of alport syndrome. Hum Gene Ther. (2019) 30:865–881. 10.1089/hum.2018.205 30808234

[51] Study of Lademirsen (SAR339375) in Patients with Alport Syndrome (HERA). Bethesda, MD: National Library of Medicine (2016). Available online at: https://clinicaltrials.gov/ct2/show/NCT02855268 (accessed February 10, 2022).

[52] Dominic C Brianna D Duane D Daniel M Gina S Jared H Get al. The dual ETAR/ATR1 blocker sparsentan slows renal disease, improves lifespan, and attenuates hearing loss in Alport mice: comparison with losartan. Nephrol Dialysis Transplant. (2020) 35:33. 10.1093/ndt/gfaa140.MO033

[53] Study of Sparsentan Treatment in Pediatrics with Proteinuric Glomerular Diseases. Bethesda, MD: National Library of Medicine (2021). Available online at: https://clinicaltrials.gov/ct2/show/NCT05003986 (accessed March 07, 2022).

[54] Trachtman H Nelson P Adler S Campbell KN Chaudhuri A Derebail VK. DUET: A Phase 2 study evaluating the efficacy safety of sparsentan in patients with FSGS. J Am Soc Nephrol. (2018). 29:2745-54. 10.1681/ASN.2018010091 30361325 PMC6218860

[55] Komers R Diva U Inrig JK Loewen A Trachtman H Rote WE. Study Design of the Phase 3 Sparsentan vs. Irbesartan (DUPLEX) study in patients with focal segmental glomerulosclerosis. Kidney Int Rep. (2020) 5:494–502. 10.1016/j.ekir.2019.12.017 32274453 PMC7136327

[56] de Zeeuw D Coll B Andress D Brennan JJ Tang H Houser M. The endothelin antagonist atrasentan lowers residual albuminuria in patients with type 2 diabetic nephropathy. J Am Soc Nephrol. (2014) 25:1083–93. 10.1681/ASN.2013080830 24722445 PMC4005314

[57] Heerspink HJL Parving HH Andress DL Bakris G Correa-Rotter R Hou FF. Atrasentan and renal events in patients with type 2 diabetes and chronic kidney disease (SONAR): a double-blind, randomised, placebo-controlled trial. Lancet. (2019) 393:1937–47. 10.1016/S0140-6736(19)30772-X 30995972

[58] Atrasentan in Patients with Proteinuric Glomerular Diseases. Bethesda, MD: National Library of Medicine (2020). Available online at: https://clinicaltrials.gov/ct2/show/NCT04573920

[59] Frida E Børge GN Marianne B. Familial hypercholesterolemia and risk of peripheral arterial disease and chronic kidney disease. J Clinic Endocrinol Metabol. (2018) 103:4491–500. 10.1210/jc.2018-01058 30085243

[60] Herman-Edelstein M Scherzer P Tobar A Levi M Gafter U. Altered renal lipid metabolism and renal lipid accumulation in human diabetic nephropathy. J Lipid Res. (2014) 55:561–72. 10.1194/jlr.P040501 24371263 PMC3934740

[61] Merscher-Gomez S Guzman J Pedigo CE Lehto M Aguillon-Prada R Mendez A. Cyclodextrin protects podocytes in diabetic kidney disease. Diabetes. (2013) 62:3817–27. 10.2337/db13-0399 23835338 PMC3806621

[62] Pedigo CE Ducasa GM Leclercq F Sloan A Mitrofanova A Hashmi T. Local TNF causes NFATc1-dependent cholesterol-mediated podocyte injury. J Clin Invest. (2016) 126:3336–50. 10.1172/JCI85939 27482889 PMC5004940

[63] Wang Z Jiang T Li J Proctor G McManaman JL Lucia S. Regulation of renal lipid metabolism, lipid accumulation, and glomerulosclerosis in FVBdb/db mice with type 2 diabetes. Diabetes. (2005) 54:2328–35. 10.2337/diabetes.54.8.2328 16046298

[64] Kang HM Ahn SH Choi P Ko YA Han SH Chinga F. Defective fatty acid oxidation in renal tubular epithelial cells has a key role in kidney fibrosis development. Nat Med. (2015) 21:37–46. 10.1038/nm.3762 25419705 PMC4444078

[65] Ge M Merscher S Fornoni A. Use of lipid-modifying agents for the treatment of glomerular diseases. J Pers Med. (2021) 11:820. 10.3390/jpm11080820 34442464 PMC8401447

[66] Jin-Ju K Sydney S Wilbon A. Podocyte lipotoxicity in CKD. Kidney. (2021) 2:755–62. 10.34067/KID.0006152020 35373048 PMC8791311

[67] Fornoni A Merscher S Kopp JB. Lipid biology of the podocyte–new perspectives offer new opportunities. Nat Rev Nephrol. (2014) 10:379–88. 10.1038/nrneph.2014.87 24861084 PMC4386893

[68] Unger RH Clark GO Scherer PE Orci L. Lipid homeostasis, lipotoxicity and the metabolic syndrome. Biochim Biophys Acta. (2010) 1801:209–14. 10.1016/j.bbalip.2009.10.006 19948243

[69] Kim JJ David JM Wilbon SS Santos JV Patel DM Ahmad A. Discoidin domain receptor 1 activation links extracellular matrix to podocyte lipotoxicity in Alport syndrome. EBioMed. (2021) 63:103162. 10.1016/j.ebiom.2020.103162 33340991 PMC7750578

[70] Mitrofanova A Molina J Varona Santos J Guzman J Morales XA Ducasa GM. Hydroxypropyl-β-cyclodextrin protects from kidney disease in experimental Alport syndrome and focal segmental glomerulosclerosis. Kidney Int. (2018) 94:1151–59. 10.1016/j.kint.2018.06.031 30301568 PMC6278936

[71] Liu X Ducasa GM Mallela SK Kim JJ Molina J Mitrofanova A. Sterol-O-acyltransferase-1 has a role in kidney disease associated with diabetes and Alport syndrome. Kidney Int. (2020) 98:1275–85. 10.1016/j.kint.2020.06.040 32739420 PMC7606642

[72] Merscher S Pedigo C Mendez A. Metabolism, energetics, and lipid biology in the podocyte – cellular cholesterol-mediated glomerular injury. Front Endocrinol. (2014) 5:169. 10.3389/fendo.2014.00169 25352833 PMC4196552

[73] Ducasa GM Mitrofanova A Fornoni A. Crosstalk between lipids and mitochondria in diabetic kidney disease. Curr Diab Rep. (2019) 19:144. 10.1007/s11892-019-1263-x 31754839 PMC12862944

[74] Ding W Yousefi K Goncalves S Goldstein BJ Sabater AL Kloosterboer A. Osteopontin deficiency ameliorates Alport pathology by preventing tubular metabolic deficits. JCI Insight. (2018) 3:e94818. 10.1172/jci.insight.94818 29563333 PMC5926939

[75] Ducasa GM Mitrofanova A Mallela SK Liu X Molina J. ATP-binding cassette A1 deficiency causes cardiolipin-driven mitochondrial dysfunction in podocytes. J Clin Invest. (2019) 129:3387–400. 10.1172/JCI125316 31329164 PMC6668702

[76] (KDIGO) CKD Work Group. KDIGO clinical practice guideline for lipid management in chronic kidney disease. Kidney Inter Suppl. (2013) 3:271–9. Available online at: https://kdigo.org/wp-content/uploads/2017/02/KDIGO-2013-Lipids-Guideline-English.pdf

[77] Esmeijer K Dekkers OM de Fijter JW Dekker FW Hoogeveen EK. Effect of different types of statins on kidney function decline and proteinuria: a network meta-analysis. Sci Rep. (2019) 9:16632. 10.1038/s41598-019-53064-x 31719617 PMC6851118

[78] Koepke ML Weber M Schulze-Lohoff E Beirowski B Segerer S Gross O. Nephroprotective effect of the HMG-CoA-reductase inhibitor cerivastatin in a mouse model of progressive renal fibrosis in Alport syndrome. Nephrol Dial Transplant. (2007) 22:1062–9. 10.1093/ndt/gfl810 17287218

[79] Crumling MA King KA Duncan RK. Cyclodextrins and iatrogenic hearing loss: new drugs with significant risk. Front Cell Neurosci. (2017) 11:355. 10.3389/fncel.2017.00355 29163061 PMC5676048

[80] Gross O Beirowski B Koepke ML Kuck J Reiner M Addicks K. Preemptive ramipril therapy delays renal failure and reduces renal fibrosis in COL4A3-knockout mice with Alport syndrome. Kidney Int. (2003) 63:438–46. 10.1046/j.1523-1755.2003.00779.x 12631109

[81] Gross O Schulze-Lohoff E Koepke ML Beirowski B Addicks K Bloch W. Antifibrotic, nephroprotective potential of ACE inhibitor vs. AT1 antagonist in a murine model of renal fibrosis. Nephrol Dial Transplant. (2004) 19:1716–23. 10.1093/ndt/gfh219 15128880

[82] Gross O Girgert R Beirowski B Kretzler M Kang HG Kruegel J. Loss of collagen-receptor DDR1 delays renal fibrosis in hereditary type IV collagen disease. Matrix Biol. (2010) 29:346–56. 10.1016/j.matbio.2010.03.002 20307660

[83] Richter H Satz AL Bedoucha M Buettelmann B Petersen AC Harmeier A. DNA-Encoded library-derived ddr1 inhibitor prevents fibrosis and renal function loss in a genetic mouse model of alport syndrome. ACS Chem Biol. (2019) 14:37–49. 10.1021/acschembio.8b00866 30452219 PMC6343110

[84] Sodek J Ganss B McKee MD. Osteopontin. Crit Rev Oral Biol Med. (2000) 11:279–303. 10.1177/10454411000110030101 11021631

[85] Li J Yousefi K Ding W Singh J Shehadeh LA. Osteopontin RNA aptamer can prevent and reverse pressure overload-induced heart failure. Cardiovasc Res. (2017) 113:633–43. 10.1093/cvr/cvx016 28453726 PMC7526752

[86] Lorenzen J Shah R Biser A Staicu SA Niranjan T Garcia AM. The role of osteopontin in the development of albuminuria. J Am Soc Nephrol. (2008) 19:884-90. 10.1681/ASN.2007040486 18443355 PMC2386721

[87] Schrezenmeier E Dörner T. Mechanisms of action of hydroxychloroquine and chloroquine: implications for rheumatology. Nat Rev Rheumatol. (2020) 16:155–66. 10.1038/s41584-020-0372-x 32034323

[88] Liu LJ Yang YZ Shi SF Bao YF Yang C Zhu SN. Effects of hydroxychloroquine on proteinuria in iga nephropathy: a randomized controlled trial. Am J Kidney Dis. (2019) 74:15–22. 10.1053/j.ajkd.2019.01.026 30922594

[89] Yang Yz Chen P Liu LJ Cai Shi SF Chen YQ. Comparison of the effects of hydroxychloroquine and corticosteroid treatment on proteinuria in IgA nephropathy: a case-control study. BMC Nephrol. (2019) 20:297. 10.1186/s12882-019-1488-6 31382914 PMC6683466

[90] Study of Hydroxychloroquine in Patients with X-linked Alport Syndrome in China (CHXLAS). Bethesda, MD: National Library of Medicine (2021). Available online at: https://clinicaltrials.gov/ct2/show/NCT04937907

[91] Omachi K Kaseda S Yokota T Kamura M Teramoto K Kuwazuru J. Metformin ameliorates the severity of experimental Alport syndrome. Sci Rep. (2021) 11:7053. 10.1038/s41598-021-86109-1 33782421 PMC8007696

[92] Teng M Wolf M Lowrie E Ofsthun N Lazarus JM Thadhani R. Survival of patients undergoing hemodialysis with paricalcitol or calcitriol therapy. N Engl J Med. (2003) 349:446–56. 10.1056/NEJMoa022536 12890843

[93] Fryer RM Rakestraw PA Nakane M Dixon D Banfor PN Koch KA. Differential inhibition of renin mRNA expression by paricalcitol and calcitriol in C57/BL6 mice. Nephron Physiol. (2007) 106:p76–81. 10.1159/000104875 17622742

[94] Rubel D Stock J Ciner A Hiller H Girgert R Müller GA. Antifibrotic, nephroprotective effects of paricalcitol vs. calcitriol on top of ACE-inhibitor therapy in the COL4A3 knockout mouse model for progressive renal fibrosis. Nephrol Dial Transplant. (2014) 29:1012–9. 10.1093/ndt/gft434 24198271

[95] Germain DP Hughes DA Nicholls K Bichet DG Giugliani R Wilcox WR. Treatment of fabry's disease with the pharmacologic chaperone migalastat. N Engl J Med. (2016) 375:545–55. 10.1056/NEJMoa1510198 27509102

[96] Bekheirnia MR Reed B Gregory MC McFann K Shamshirsaz AA Masoumi A. Genotype-phenotype correlation in X-linked Alport syndrome. J Am Soc Nephrol. (2010) 21:876–83. 10.1681/ASN.2009070784 20378821 PMC2865738

[97] Savige J Storey H Il Cheong H Gyung Kang H Park E Hilbert P. X-Linked and autosomal recessive alport syndrome: pathogenic variant features and further genotype-phenotype correlations. PLoS One. (2016) 11:e0161802. 10.1371/journal.pone.0161802 27627812 PMC5023110

[98] Zhang Y Böckhaus J Wang F Wang S Rubel D Gross O. Genotype-phenotype correlations and nephroprotective effects of RAAS inhibition in patients with autosomal recessive Alport syndrome. Pediatr Nephrol. (2021) 36:2719–30. 10.1007/s00467-021-05040-9 33772369 PMC8370956

[99] Wang D Mohammad M Wang Y Tan R Murray LS Ricardo S. The chemical chaperone, pba, reduces er stress and autophagy and increases collagen iv α5 expression in cultured fibroblasts from men with x-linked alport syndrome and missense mutations. Kidney Int Rep. (2017) 2:739–48. 10.1016/j.ekir.2017.03.004 29142990 PMC5678609

[100] Jones FE Murray LS McNeilly S Dean A Aman A Lu Y. 4-Sodium phenyl butyric acid has both efficacy and counter-indicative effects in the treatment of Col4a1 disease. Hum Mol Genet. (2019) 28:628–38. 10.1093/hmg/ddy369 30351356 PMC6360271

[101] Li H Yang Y Hong W Huang M Wu M Zhao X. Applications of genome editing technology in the targeted therapy of human diseases: mechanisms, advances and prospects. Signal Transduct Target Ther. (2020) 5:1. 10.1038/s41392-019-0089-y 32296011 PMC6946647

[102] Cox D Platt R Zhang F. Therapeutic genome editing: prospects and challenges. Nat Med. (2015) 21:121–31. 10.1038/nm.3793 25654603 PMC4492683

[103] Luther DC Lee YW Nagaraj H Scaletti F Rotello VM. Delivery approaches for CRISPR/Cas9 therapeutics *in vivo*: advances and challenges. Expert Opin Drug Deliv. (2018) 15:905–13. 10.1080/17425247.2018.1517746 30169977 PMC6295289

[104] Quinlan D Catherine R. Genetic Basis of Type IV collagen disorders of the kidney. CJASN. (2021) 16:1101–9. 10.2215/CJN.19171220 33849932 PMC8425620

[105] WareJoncas Z Campbell JM Martínez-Gálvez G Gendron WAC Barry MA Harris PC. Precision gene editing technology and applications in nephrology. Nat Rev Nephrol. (2018) 14:663–77. 10.1038/s41581-018-0047-x 30089813 PMC6591726

[106] Daga S Donati F Capitani K Croci K Tita R Giliberti A. New frontiers to cure Alport syndrome: *COL4A3* and *COL4A5* gene editing in podocyte-lineage cells. Eur J Hum Genet. (2020) 28:480–90. 10.1038/s41431-019-0537-8 31754267 PMC7080842

[107] Lin X Suh JH Go G Miner JH. Feasibility of repairing glomerular basement membrane defects in Alport syndrome. J Am Soc Nephrol. (2014) 25:687–92. 10.1681/ASN.2013070798 24262794 PMC3968506

[108] Funk SD Bayer RH Miner JH. Endothelial cell-specific collagen type IV-α_3_ expression does not rescue Alport syndrome in Col4a3^−/−^ mice. Am J Physiol Renal Physiol. (2019) 316:F830–7. 10.1152/ajprenal.00556.2018 30724107 PMC6580247

[109] Heikkilä P Tibell A Morita T Chen Y Wu G Sado Y. Adenovirus-mediated transfer of type IV collagen alpha5 chain cDNA into swine kidney *in vivo*: deposition of the protein into the glomerular basement membrane. Gene Ther. (2001) 8:882–90. 10.1038/sj.gt.3301342 11423936

[110] Petropoulos S Edsgärd D Reinius B Deng Q Panula SP Codeluppi S. Single-Cell RNA-Seq reveals lineage and x chromosome dynamics in human preimplantation embryos. Cell. (2016) 167:285. *Erratum for: Cell. (2016). 165:1012-26*. 10.1016/j.cell.2016.03.023 27062923 PMC4868821

[111] Kuebler B Aran B Miquel-Serra L Muñoz Y Ars E Bullich G. Integration-free induced pluripotent stem cells derived from a patient with autosomal recessive Alport syndrome (ARAS). Stem Cell Res. (2017) 25:1–5. 10.1016/j.scr.2017.08.021 29246570

[112] Sugimoto H Mundel TM Sund M Xie L Cosgrove D Kalluri R. Bone-marrow-derived stem cells repair basement membrane collagen defects and reverse genetic kidney disease. Proc Natl Acad Sci U S A. (2006) 103:7321–6. 10.1073/pnas.0601436103 16648256 PMC1464339

[113] Moschidou D Corcelli M Hau KL Ekwalla VJ Behmoaras JV De Coppi P. Human chorionic stem cells: podocyte differentiation and potential for the treatment of alport syndrome. Stem Cells Dev. (2016) 25:395–404. 10.1089/scd.2015.0305 26728561

[114] Ninichuk V Gross O Segerer S Hoffmann R Radomska E Buchstaller A. Multipotent mesenchymal stem cells reduce interstitial fibrosis but do not delay progression of chronic kidney disease in collagen4A3-deficient mice. Kidney Int. (2006) 70:121–9. 10.1038/sj.ki.5001521 16723981

